# Retraction: ALK3-SMAD1/5 signaling mediates the BMP2-induced decrease in PGE2 production in human endometrial stromal cells and decidual stromal cells

**DOI:** 10.3389/fcell.2026.1988449

**Published:** 2026-09-10

**Authors:** Frontiers Editorial Office

The journal retracts the 15 September 2020 article cited above.

Following publication, concerns were raised on PubPeer regarding the integrity of the images in the published figures. Specifically, Figure 6D a-Tubulin control lanes 1–3 appear identical to Figure 7B for a-tubulin control lanes 2–4.

The authors failed to provide a satisfactory explanation during the investigation, which was conducted in accordance with Frontiers’ policies. As a result, the data and conclusions of the article have been deemed unreliable and the article has been retracted.

This retraction was approved by the Chief Executive Editor of Frontiers. The authors do not agree to this retraction.

Frontiers would like to thank Claire Francis for contacting the journal regarding the published article.

